# Biotechnological and Biomedical Applications of Enzymes Involved in the Synthesis of Nucleosides and Nucleotides

**DOI:** 10.3390/biom11081147

**Published:** 2021-08-03

**Authors:** Jesús Fernández-Lucas

**Affiliations:** 1 Applied Biotechnology Group, Biomedical Science School, Universidad Europea de Madrid, 28670 Villaviciosa de Odón, Spain; jesus.fernandez2@universidadeuropea.es; 2 Research Group in Natural and Exact Sciences, GICNEX, Universidad de la Costa, CUC, Barranquilla 080002, Colombia

Nucleic acid derivatives are involved in cell growth and replication, but they are also particularly important as building blocks for RNA and DNA synthesis. In nature, purine and pyrimidine nucleotides are synthesized through two distinct pathways, de novo and salvage pathways, both depending on 5-phospho-α-D-ribose 1-diphosphate (PRPP) as a key element [1,2]. In the de novo pathway, purine and pyrimidine nucleotides are synthesized from simple molecules such as glycine, glutamine, or aspartate. In contrast, the salvage pathway employs scavenged preformed endogenous or exogenous nucleobases to generate the corresponding nucleoside-5′-monophosphates (NMPs) [3]. Both metabolic routes, de novo and salvage pathways, lead to the synthesis of NMPs, which are subsequently phosphorylated to obtain the corresponding nucleoside-5′-di (NDPs) and triphosphates (NTPs). Moreover, all organisms also generate (2′-deoxy)nucleoside-5′-diphosphates (dNDPs) from NDPs [4], which will be converted to 2′-deoxyribonucleotides (dNTPs), as precursors for DNA synthesis. Additionally, nucleotide derivatives are involved in cell signaling (cyclic nucleotides, cNMPs or c-di-NMPs) [5] and a multitude of different biochemical processes, acting as cofactors (NADP^+^) or energy sources (ATP).

Due to the importance of the enzymes involved in the synthesis of nucleosides and nucleotides, they have been extensively studied as potential targets for chemotherapy against different diseases [1,6] or as important biocatalysts for the synthesis of different nucleoside and nucleotide analogs [2,7].

This Special Issue will cover the most recent and relevant findings concerning biotechnological and biomedical applications of these enzymes. To this end, a total of seven experimental articles written by world-leading experts were compiled to show the state-of-the-art.

In the biocatalysis section, glycosyltransferases are the most cited enzymes, and several authors focus their attention on this enzyme family. In this respect, Kayushin et al. [8] developed the preparative synthesis of 2-fluorocordycepin from 2-fluoroadenosine and 3′-deoxyinosine catalyzed by purine nucleoside phosphorylase from *E. coli* but also explored the presence of by-products in this process, providing new insights about this undesirable effect. Similarly, Rivero et al. performed the sustainable production of cladribine (an FDA-approved anticancer agent) by a novel 2′-deoxyribosyltransferase (NDT)-based biocatalyst, including a covalent immobilization of *Lactobacillus delbrueckii* 2′-deoxyribosyltransferase (*Ld*NDT) onto glutaraldehyde-activated biomimetic silica nanoparticles followed by biocatalyst entrapment in calcium alginate [9]. Finally, similar to that shown above, other authors displayed the importance of transglycosylation reactions in biocatalysis by exploring the differences of the enzymatic and chemical syntheses of vacor analogs of nicotinamide riboside, NMN, and NAD [10].

Moreover, enzymatic cascades play an essential role in this Special Issue, with three different articles about this topic. In the first one, a study of the possibilities of ribokinase (RK)/phosphopentomutase (PPM)/nucleoside phosphorylase (NP) cascades in the synthesis of different modified nucleosides was carried out by comparing enzymes from mesophilic and thermophilic organisms [11]. In another interesting article, Frisch et al. developed a novel one-pot multi-enzymatic system for the synthesis of 2′-deoxynucleoside-5′-triphosphates [12]. To this end, an enzymatic cascade comprised of adenine phosphoribosyltransferase (APRT), polyphosphate kinase (PPK), and ribonucleotide reductase (RNR) was successfully employed for the synthesis of cladribine triphosphate from 2-chloroadenine and 5-phospho-α-D-ribose 1-diphosphate (PRPP) [11]. Finally, to conclude this subsection, Becker and colleagues [13] developed a multi-enzymatic cascade for the production of 2′,3′-cGAMP. This four-enzyme cascade is formed by the sequential action of adenosine kinase from *Saccharomyces cerevisiae* (*Sc*ADK), polyphosphate kinase 2 from *Acinetobacter johnsonii* (*Aj*PPK2), PPK2 from *Sinorhizobium meliloti* (*Sm*PPK2), and a truncated human cyclic GMP-AMP synthase (t*Hs*cGAS). Interestingly the action of the coupled system *Sc*ADK/*Aj*PPK2/*Sm*PPK2 led to the three-step synthesis of ATP from adenosine, which was subsequently transformed to GMP-AMP (2′,3′-cGAMP) by the coupled t*Hs*cGAS.

Regarding the biomedical applications, Acosta et al. reported the first application of a 2′-deoxyribosyltransferase (NDT) in suicide gene therapy for cancer treatment, and also deep into the molecular mechanisms of the hydrolytic ability of *Ld*NDT over different nucleoside-based prodrugs through molecular dynamics simulations. Finally, the *Ld*NDT/2′-deoxy-2-fluoroadenosine (dFAdo) system was assayed in human cervical cancer (HeLa) cells with inhibitory concentrations in the low micromolar range [14].

Finally, we would like to thank all the authors, reviewers, and the Editorial Board members of Biomolecules for their considerable contributions to supporting the implementation of this special research topic, and we hope that the readers will enjoy their work.
